# The SolarMal Project: innovative mosquito trapping technology for malaria control

**DOI:** 10.1186/1475-2875-11-S1-O45

**Published:** 2012-10-15

**Authors:** Alexandra Hiscox, Nicolas Maire, Ibrahim Kiche, Mariabeth Silkey, Tobias Homan, Prisca Oria, Collins Mweresa, Bruno Otieno, Margaret Ayugi, Teun Bousema, Patrick Sawa, Jane Alaii, Thomas Smith, Cees Leeuwis, Wolfgang R Mukabana, Willem Takken

**Affiliations:** 1 Laboratory of Entomology, Wageningen University and Research Centre, Wageningen, The Netherlands; 2 Department of Epidemiology and Public Health, Swiss Tropical and Public Health Institute, Basel, Switzerland; 3 International Centre of Insect Physiology and Ecology, Nairobi, Kenya; 4 Department of Medical Microbiology, Radboud University, Nijmegen, The Netherlands; 5 Context Factor Solutions, Nairobi, Kenya; 6 Knowledge, Innovation and Technology Group, Wageningen University and Research Centre, Wageningen, The Netherlands

## 

The use of insecticides against mosquitoes, and drugs to treat infection, continue to form the mainstays of malaria control programmes, but the long term success and sustainability of these approaches is threatened by the development of insecticide and drug resistance. New complementary approaches to control must be explored.

The development by Okumu and others [1] of a blend of synthetic chemical attractants which was capable of attracting more *Anopheles gambiae s.s.* than a human, provided the key breakthrough towards creation of a mass trapping system which could be used for malaria control. By luring *Anopheles* mosquitoes to traps in numbers that are high enough to suppress population size and reduce biting intensity, a decline in malaria transmission could be realized. Here we describe our plans for the development and testing of odour-baited traps for malaria control in Western Kenya.

The SolarMal project aims to demonstrate proof of principle for the elimination of malaria from Rusinga Island, Western Kenya, using the nationwide adopted strategy of LLINs and case management, augmented by mass trapping of mosquito vectors. The use of novel technology and scientific development underpins all areas of the project; from the optimisation of chemical baits to attract mosquitoes, to the design of a new mosquito trap and the installation of solar panel systems to provide power to run the traps. Electronic tablets are used to record health and demographic surveillance data.

The mosquito traps operate according to a counterflow mechanism previously shown to be highly effective in collecting anopheline mosquitoes [2] and are designed to collect mosquitoes outdoors prior to house entry. Odour baits placed within the traps mimic human odourants [3].

In a unique variation on the stepped wedge intervention strategy, which we refer to as the hierarchical design, intervention implementation begins at one randomly selected household and expands radially until a cluster of houses with the intervention is created. The intervention implementation then commences in a second geographically distinct location, then a third, fourth, fifth etc, continuing until the whole island is covered.

Outcome measures of malaria parasite prevalence and incidence, as well as estimates of malaria transmission intensity, will be used to assess the impact of the intervention. We expect the results to demonstrate that the use of odour baited traps is an effective, novel means of integrated malaria control.
